# The Effects of Syphilis Infection on Total Knee Arthroplasty Outcomes: A Retrospective Cohort Study

**DOI:** 10.3390/jcm13237116

**Published:** 2024-11-25

**Authors:** Paul Gudmundsson, Marc Gadda, Aruni Areti, Senthil Sambandam

**Affiliations:** 1 UT Southwestern Medical Center, 5323 Harry Hines Blvd, Dallas, TX 75390, USA; 2 Baylor College of Medicine, 1 Baylor Plz, Houston, TX 77030, USA

**Keywords:** total knee arthroplasty, syphilis, sexually transmitted diseases, surgical outcomes, postoperative complications

## Abstract

**Objective:** This study investigated the impact of recent syphilis infection on postoperative outcomes following total knee arthroplasty (TKA). We hypothesized that patients with a documented history of syphilis infection would experience a higher rate of postoperative complications compared to those without such a history. **Methods:** We conducted a retrospective cohort analysis using a national insurance claims database. Our study population included 237,360 patients who underwent primary TKA between 2005 and 2024. Patients were classified into two groups based on the presence (+Syph) or absence (−Syph) of a syphilis diagnosis within one year prior to the TKA. We evaluated the rates of several postoperative complications at 30 days postsurgery, including infection, hematologic issues, and cardiac events. Statistical analyses between groups was performed using chi-squared tests and Fisher’s exact tests. Routine demographic data such as age, sex, race, and comorbidities were also analyzed. **Results:** Among the 237,360 TKA patients, we identified 71 with a history of syphilis within one year of their surgery. The +Syph group exhibited significantly higher rates of periprosthetic infection (4.23% vs. 0.81%, *p* = 0.001), need for manipulation under anesthesia (MUA) at four months (7.04% vs. 2.82%, *p* = 0.032), deep venous thrombosis (4.23% vs. 1.27%, *p* = 0.026), periprosthetic fracture (2.82% vs. 0.23%, *p* < 0.001), and pneumonia (2.82% vs. 0.62%, *p* = 0.019) within 30 days postTKA. No significant differences were observed in 30-day mortality, deep or superficial surgical site infections, wound dehiscence, blood loss anemia, or transfusion requirements. Additionally, rates of acute renal failure, pulmonary embolism, and cardiac events did not differ significantly between groups. Demographically, patients in the syphilis cohort had a higher prevalence of smoking and diabetes preoperatively within one year of their surgical date. **Conclusions:** A documented syphilis diagnosis within one year of TKA significantly affects postoperative outcomes, increasing the rates of prosthetic joint infection, MUA, deep venous thrombosis, periprosthetic fracture, and pneumonia. These findings underscore the need for heightened vigilance in the pre- and postoperative management of patients with a history of syphilis infection undergoing TKA. Further research is warranted to explore the relationship between prior syphilis infection and TKA outcomes, as well as to develop strategies to mitigate this increased risk.

## 1. Introduction

Total knee arthroplasty (TKA) is a widely performed surgical intervention aimed at relieving pain and improving function in patients with severe knee osteoarthritis and other debilitating knee conditions. In the United States, the prevalence of TKA has been increasing significantly, with approximately 800,000 to 1 million procedures conducted annually [1]. This trend reflects both an aging population and a growing acceptance of the procedure, with projections suggesting that the number of TKA procedures may exceed 1.2 million by 2030 due to rising obesity rates and an aging demographic [2].

Syphilis, a chronic infectious disease caused by *Treponema pallidum*, has also seen a notable resurgence in the U.S. In 2022, the incidence of primary and secondary syphilis reached approximately 176,500 cases, translating to a rate of about 52.0 cases per 100,000 population [3]. This increase is particularly pronounced among high-risk groups, including men who have sex with men (MSM) and individuals living with HIV [4]. Recent advancements in diagnostic methods, such as nucleic acid amplification tests (NAATs), have improved early detection and management of syphilis. However, traditional serological assays remain vital for diagnosis and monitoring [5]. Benzathine penicillin G remains the first-line therapy for all stages of syphilis, although alternative regimens are available for those with penicillin allergies [6].

The intersection of syphilis and TKA introduces unique challenges. Syphilis can affect various aspects of TKA, including preoperative assessment, surgical outcomes, and postoperative recovery. Syphilis infection can impair immune function and cause vascular disease, which can both influence healing, infection, and the development of postoperative complications. Furthermore, the systemic effects of tertiary syphilis, such as cardiovascular and neurological complications, necessitate comprehensive preoperative evaluations to ensure surgical safety [7]. While direct evidence linking syphilis to specific TKA complications is limited, the potential for increased risks, such as wound healing issues and postoperative infections due to the infection’s systemic effects is a significant concern [8].

Understanding the effects of syphilis on TKA outcomes is crucial for optimizing patient management. This study aimed to evaluate the impact of syphilis on the outcomes of patients that undergo TKA and to expand upon the relative paucity of literature on this specific patient population. By examining the intersection of these two important and increasingly prevalent health issues, this research seeks to enhance clinical practices and improve outcomes for patients undergoing TKA with a history of syphilis infection.

## 2. Methods

### 2.1. Data Sourcing

The TriNetX Research network (https://trinetx.com, Cambridge, MA, USA) was utilized for this retrospective cohort study. Data were collected from this database of over 120 million patient records from 82 healthcare organizations (HCOs) participating in the TriNetX research network. This includes inpatient, outpatient, and emergency department data from institutions such as hospitals, health systems, and academic medical centers. TriNetX aggregates data from sources such as electronic health records and payer information and provides advanced tools for querying patient cohorts and analyzing data.

### 2.2. Cohort Construction

Patients aged 18 and older who underwent primary TKA between 2005 and 2024 were identified using CPT code 27447. A syphilis diagnosis was defined using the ICD-9 and ICD-10 codes listed in Appendix A. The +Syph group comprised patients with a documented ICD9 and ICD 10 code for syphilis within one year prior to TKA, while the −Syph group included patients without a documented ICD code for syphilis within that same timeframe. Baseline demographic data were collected and included sex, race, marital status, age, history of diabetes, obesity, and smoking. Patients were not matched based on baseline demographics. Patient records missing any of the data categories examined in this study were excluded from analysis. Surgical and medical complications were identified using the ICD and CPT codes listed in Appendix A.

### 2.3. Index Event and Outcome Analysis

This study assessed several peri- and postoperative complications, including 30-day mortality, deep and superficial surgical site infection, wound dehiscence, periprosthetic infection, manipulations under anesthesia, acute renal failure, blood loss anemia, cardiac arrest, deep venous thrombosis, periprosthetic mechanical complications, pulmonary embolism, periprosthetic fracture, pneumonia, stroke, and transfusion requirements. The date of TKA was designated as the index event from which the follow-up period began. All outcomes were compared at a postoperative follow-up of 30-days, except for the complication of a need for manipulation under anesthesia, which was measured at four months postoperatively. Information about the outcomes of interest is provided in Appendix A.

### 2.4. Statistical Analysis

Odds ratios were calculated to compare the rates of complications between the exposure and control groups, and 95% confidence intervals were provided for all calculations. Preoperative variables were compared using chi-squared analysis, and Fisher’s exact test was utilized when the cell count was less than 5. Postoperative complications were compared using Chi-squared analysis (Fisher exact test when less than 5). *p* < 0.05 was considered statistically significant with two-sided tests.

### 2.5. Software Employed for Statistical Analysis and Data Visualization

The TriNetx Live platform was used for data compilation. SPSS version 29 (IBM) was used for data management and statistical analysis. Microsoft Excel was used for further statistical analysis and data visualization. The corresponding author (SS) independently verified the data accuracy and statistical analysis.

### 2.6. Data Integrity and Ethical Considerations

The TriNetX platform adheres to HIPAA and GDPR regulations to ensure data security and protect patient privacy as it contains solely de-identified aggregate information. This study was exempt from Institutional Review Board approval, as it utilized de-identified data and did not involve direct human subjects.

## 3. Results

### 3.1. Demographics

A total of 237,360 patients who underwent TKA between 2005 and 2024 were identified, of whom 71 had a history of syphilis within one year prior to their surgical date (Table 1). The demographic data were compared between the two groups (Table 2). The +Syph TKA group included a higher proportion of males (45.5% vs. 39%) and patients younger than 65 years (47.9% vs. 38.5%). The +Syph group had a higher proportion of American Indian of Alaska Native (2.82% vs. 0.27%), Black or African American (23.94% vs. 9.73%), and Other race (2.82% vs. 2.43%) patients compared to the −Syph group (*p* < 0.001). In terms of medical comorbidities, the +Syph group consisted of a higher proportion of individuals with a prior diagnosis of diabetes (33.8%. vs. 24.8%, *p* = 0.003) and smokers (38.0% vs. 19.8%, *p* < 0.001) compared to the −Syph TKA group.

### 3.2. 30-Day Postoperative Outcomes

Patients in the +Syph group exhibited significantly higher rates of several postoperative complications within 30 days following TKA (Table 3). These complications included periprosthetic infection (4.23% vs. 0.81%, *p* = 0.001), deep venous thrombosis (4.23% vs. 1.27%, *p* = 0.026), periprosthetic fracture (2.82% vs. 0.23%, *p* < 0.001), and pneumonia (2.82% vs. 0.62%, *p* = 0.019). There were no significant differences between the two groups regarding 30-day mortality, deep or superficial surgical site infections (SSIs), wound dehiscence, blood loss anemia, transfusion requirements, or other medical complications, including acute renal failure, pulmonary embolism, or cardiac events.

### 3.3. Risk of Manipulation Under Anesthesia

A separate analysis for the complication of a need for manipulation under anesthesia (MUA) was performed at a different time point of four months postoperation. We found a significantly higher risk of this complication in the +Syph cohort compared to the −Syph group (7.04% vs. 2.82%, *p* = 0.032).

## 4. Discussion

This is the first study to our knowledge that has examined the impact of recent syphilis infection on recovery from TKA, and the results suggest that this comorbidity has a measurable effect on postoperative outcomes. Our data reveal significantly higher rates of several postoperative complications following TKA in the +Syph cohort compared to those without a history of this infection. These findings underscore the importance of considering a patient’s infectious and sexual behavioral history when evaluating eligibility for this elective procedure.

While the +Syph cohort did not experience higher rates of all complication types examined in this paper, there were several categories of poor outcomes that were more common compared to their −Syph counterparts. Specifically, the +Syph cohort demonstrated significantly higher rates of periprosthetic infection, deep venous thrombosis (DVT), periprosthetic fracture, and postoperative pneumonia within 30 days following TKA. Although no prior literature specifically addresses the impact of syphilis infection on orthopedic surgical outcomes, our findings are consistent with the increased risk profiles associated with other sexually transmitted infections, such as HIV [9,10,11].

In addition to the results described above, the +Syph cohort was also seen to have an increased likelihood of requiring manipulation under anesthesia (MUA) at a four month follow-up. MUA is a procedure used to treat arthrofibrosis, a complication that is due to pathologic stiffening of the knee joint that can occur following TKA. It is thought that this disease process is caused by an exaggerated inflammatory response to the surgery, and it is most effectively managed through prevention with patient education, adherence to postoperative physical therapy, and controlling postoperative inflammation [12]. Although this study does not directly examine why this outcome is more common in the +Syph group, it could be related to factors such as patient compliance with pre- and postoperative protocols or the systemic inflammation associated with the infection.

Despite the increased rates of certain complications in the +Syph group, many other negative surgical outcomes did not differ significantly from the control group. Between the two cohorts, there were no observed differences in 30-day mortality, deep or superficial SSI, wound dehiscence, blood loss anemia or transfusion requirements. Additionally, there were no significant differences in rates of acute renal failure, pulmonary embolism, or cardiac events. These findings indicate that, despite the elevated risk profile associated with syphilis infection, it does not adversely affect all surgical outcomes. TKA can still be a safe procedure in this cohort, and syphilis infection alone should not be a strict contraindication for this elective procedure.

This study did not explore the underlying mechanisms behind the increased complication rates observed in the +Syph group. Potential contributing factors may include systemic inflammation due to the infection, co-infection with diseases like HIV, and its association with other known surgical risk factors such as diabetes, smoking, drug use and lower socioeconomic status. Our data found patients in the +Syph cohort were significantly more likely to be male, diabetic, and have a history of smoking, which is consistent with prior literature. Gomes et al. reported higher rates of syphilis in males, those with low educational attainment, and single marital status, along with additional behavioral factors, including men who have sex with men, drug users, and individuals with a history of STDs in the past year [13]. The prevalence of these comorbidities adds complexity to our findings, suggesting that the impact of syphilis on TKA outcomes may be influenced by these factors. Future research should aim to account for these confounders through multivariate analysis to isolate the specific effects of recent syphilis infection on TKA surgical results separately from the potential influence of confounding factors.

Although there are multiple potential factors that may influence the results of this study, one particular confounder that should be mentioned is the possibility of co-infection with HIV. It has been estimated that at least 15% of patients in the United States with a syphilis infection are co-infected with HIV [14,15]. The effects of HIV infection on arthroplasty outcomes are well-studied, and an association of HIV infection with outcomes such as increased hospital stay, perioperative wound complications, and prosthetic joint infection has been demonstrated [16,17]. This potential impact of HIV coinfection may explain some of the effects seen in this study, and future research is needed to control for this confounding factor.

The results of this study have several clinical implications. First, they suggest that, despite higher rates of certain complications in this population, the absolute number of complications remains within an acceptable range, indicating that TKA is a clinically safe procedure for these patients. Routine preoperative screening for syphilis and other sexually transmitted infections could help identify at-risk patients prior to surgery. Early identification may facilitate improved preoperative counseling and postoperative care, potentially enhancing outcomes for these individuals.

## 5. Limitations

This study has several limitations. First, as a retrospective study with data sourced from a large database, data integrity lies outside the control of the investigators. It was not possible to screen all patients for syphilis or ensure adequate follow-up, potentially affecting the reported rates of infection and the surgical outcomes. Specific to the TriNetX database, the data are limited to generic ICD and CPT codes without access to raw, patient-specific information. This leads to an inability to match for comorbidities, which could also introduce confounding variables. Without direct access to patient-level data, data quality cannot be fully validated.

In addition to the aforementioned limitations of the data used for this study, there are aspects of the study design that limit the results. As a retrospective study with a single follow-up point, it does not provide insights into the longitudinal history of this patient population or the potential for long-term complications. The 30-day follow-up period used for this study may not capture all relevant postoperative complications, especially those such as infections or mechanical failures that commonly manifest later in the postoperative period. Further research can expand on the results of this study to better characterize the long-term risks of TKA in this cohort.

Finally, the retrospective study design of this paper also does not allow for the establishment of a cause and effect relationship between syphilis infection and complications following TKA. Future prospective studies with larger sample sizes are essential to thoroughly investigate the potential causal effects of syphilis infection on TKA outcomes. Moreover, further research is needed to explore the pathophysiological mechanisms linking recent syphilis infection to an increased risk of surgical complications after TKA.

## 6. Conclusions

A history of syphilis infection within one year of TKA has a statistically significant association with certain surgical outcomes, specifically with an increased risk of several acute complications. However, while these findings emphasize the need for increased vigilance in the perioperative management of affected patients, they do not imply that recent syphilis infection should serve as a strict contraindication for this elective orthopedic procedure. Future research can further characterize the risk profile of these patients and guide the development of protocols to optimally manage this unique population.

## Figures and Tables

**Table 1:** Total number of patients in the +Syph and −Syph groups.

Group	Number of Patients
Total patients	237,360
+Syph	71
−Syph	237,289

**Table 2:** Patient demographics and medical comorbidities of the +Syph and −Syph groups.

Patient Demographics and Admission Characteristics of +Syph and −Syph Groups				
Variables		+Syph (*n* = 71)	−Syph (*n* = 237,289)	Significance
Age > 65		34 (47.89%)	91,385 (38.51%)	0.105
Sex (female proportion)		37 (52.11%)	138,969 (58.57%)	0.270
Obesity		29 (40.85%)	82,384 (34.72%)	0.278
Smoking disorder		27 (38.03%)	47,044 (19.83%)	<0.001 *
Diabetes without complications		24 (33.80%)	47,164 (19.88%)	0.003 *
				
Race				
	American Indian or Alaska Native	2 (2.82%)	642 (0.27%)	<0.001 *
	Asian	0 (0.00%)	4901 (2.07%)	<0.001 *
	Black or African American	17 (23.94%)	23,084 (9.73%)	<0.001 *
	Native Hawaiian or Other Pacific Islander	0 (0.00%)	479 (0.20%)	<0.001 *
	Other race	2 (2.82%)	5769 (2.43%)	<0.001 *
	Unknown	9 (12.68%)	24,818 (10.46%)	<0.001 *
	White	41 (57.75%)	177,596 (74.84%)	<0.001 *
	Hispanic or Latino	5 (7.04%)	12,339 (5.20%)	0.763
Marital status				
	Married	12 (16.90%)	70,498 (29.71%)	0.059
	Single	15 (21.13%)	39,993 (16.85%)	0.059
	Unknown	44 (61.97%)	126,798 (53.44%)	0.059

* = significant value.

**Table 3:** Postoperative complication rates, odds ratio, confidence interval and *p*-value for the +Syph and −Syph groups.

Unmatched Analysis of Postoperative Complications: Differences Between Characteristics of +Syph and −Syph Groups					
Postoperative Variables	+Syph (n = 71)	−Syph (n = 237,289)	Odds Ratio (CU/NCU Group)	Odds Ratio 95% Confidence Interval	Significance
30-Day mortality	0 (0.00%)	353 (0.15%)	-	-	0.745
Deep SSI	0 (0.00%)	28 (0.01%)	-	-	0.927
Wound dehiscence	1 (1.41%)	1245 (0.52%)	2.708	0.376–19.513	0.303
Periprosthetic infection	3 (4.23%)	1923 (0.81%)	5.400	1.698–17.176	<0.001 *
Superficial SSI	0 (0.00%)	215 (0.09%)	-	-	0.800
Manipulation under anesthesia	5 (7.04%)	6694 (2.82%)	2.610	1.051–6.480	0.032 *
Acute renal failure	2 (2.82%)	4672 (1.97%)	1.443	0.354–5.888	0.607
Blood loss anemia	8 (11.27%)	15,528 (6.54%)	1.814	0.869–3.785	0.108
Cardiac arrest and VF	0 (0.00%)	107 (0.05%)	-	-	0.858
DVT	3 (4.23%)	3010 (1.27%)	3.434	1.080–10.919	0.026 *
Periprosthetic mechanical complication	4 (5.63%)	835 (0.35%)	16.906	6.151–46.469	<0.001 *
Myocardial infarction	0 (0.00%)	501 (0.21%)	-	-	0.698
Pulmonary embolism	0 (0.00%)	1998 (0.84%)	-	-	0.437
Periprosthetic fracture	2 (2.82%)	556 (0.23%)	12.341	3.018–50.464	<0.001 *
Pneumonia	2 (2.82%)	1473 (0.62%)	4.640	1.137–18.946	0.019 *
Stroke	4 (5.63%)	6410 (2.70%)	2.150	0.784–5.899	0.128
Blood transfusion	0 (0.00%)	105 (0.04%)	-	-	0.859

* = significant value. - = Insufficient data or event occurrence to calculate an odds ratio or confidence interval.

## Data Availability

The data that support the findings of this study are available from the corresponding author, SS, upon reasonable request.

## Appendix A

### Appendix A.1. CPT Codes for Procedures

TKA: 27447

MUA: 27570

Syphilis: 090, 0901, 0902, 0903, 0904, 0905, 0906, 0907, 0908, 0909, 091, 0910, 0911, 0912, 0913, 0914, 0915, 0916, 0917, 0918, 0919, 092, 0920, 0929, 093, 0930, 0931, 0932, 0938, 0939, 095, 0950, 0951, 0952, 0953, 0954, 0955, 0956, 0957, 0958, 0959, 096, 097, 0970, 0971, 0977, 0979, A50, A500, A5001, A5002, A5003, A5004, A5005, A5006, A5007, A5008, A5009, A501, A502, A503, A5030, A5031, A5032, A5039, A504, A5040, A5041, A5042, A5043, A5044, A5045, A5049, A505, A5051, A5052, A5053, A5054, A5055, A5056, A5057, A5059, A506, A507, A509, A51, A510, A511, A512, A513, A5131, A5132, A5139, A514, A5141, A5142, A5143, A5144, A5145, A5146, A5149, A515, A519, A52, A520, A5200, A5201, A5202, A5203, A5204, A5205, A5206, A5209, A521, A5210, A5211, A5212, A5213, A5214, A5215, A5216, A5217, A5219, A522, A523, A527, A5271, A5272, A5273, A5274, A5275, A5276, A5277, A5278, A5279, A528

### Appendix A.2. Complications and Corresponding ICD 9 and 10 Codes

Stroke: 436, 4370, 4371, 4380, 43810, 43811, 43812, 43813, 43814, 43819, 43820, 43821, 43822, 43830, 43831, 43832, 43840, 43841, 43842, 43850, 43851, 43852, 43853, 4386, 4387, 43881, 43882, 43883, 43884, 43885, 43889, 4389, V1254, Z8673, G450, G451, G452, G453, G454, G458, G459, G460, G462, G463, G464, G465, G466, G467, G468, I6300, I63011, I63012, I63013, I63019, I6302, I63031, I63032, I63033, I63039, I6309, I6310, I63111, I63112, I63113, I63119, I6312, I63131, I63132, I63133, I63139, I6319, I6320, I63211, I63212, I63213, I63219, I6322, I63231, I63232, I63233, I63239, I6329, I6330, I63311, I63312, I63313, I63319, I63321, I63322, I63323, I63329, I63331, I63332, I63333, I63339, I63341, I63342, I63343, I63349, I6339, I6340, I63411, I63412, I63413, I63419, I63421, I63422, I63423, I63429, I63431, I63432, I63433, I63439, I63441, I63442, I63443, I63449, I6349, I6350, I63511, I63512, I63513, I63519, I63521, I63522, I63523, I63529, I63531, I63532, I63533, I63539, I63541, I63542, I63543, I63549, I6359, I636, I638, I6381, I6389, I639, I6930, I6931, I69310, I69311, I69312, I69313, I69314, I69315, I69318, I69319, I69320, I69321, I69322, I69323, I69328, I69331, I69332, I69333, I69334, I69339, I69341, I69342, I69343, I69344, I69349, I69351, I69352, I69353, I69354, I69359, I69361, I69362, I69363, I69364, I69365, I69369, I69390, I69391, I69392, I69393, I69398, I6980, I6981, I69810, I69811, I69812, I69813, I69814, I69815, I69818, I69819, I69820, I69821, I69822, I69823, I69828, I69831, I69832, I69833, I69834, I69839, I69841, I69842, I69843, I69844, I69849, I69851, I69852, I69853, I69854, I69859, I69861, I69862, I69863, I69864, I69865, I69869, I69890, I69891, I69892, I69893, I69898, I6990, I6991, I69910, I69911, I69912, I69913, I69914, I69915, I69918, I69919, I69920, I69921, I69922, I69923, I69928, I69931, I69932, I69933, I69934, I69939, I69941, I69942, I69943, I69944, I69949, I69951, I69952, I69953, I69954, I69959, I69961, I69962, I69963, I69964, I69965, I69969, I69990, I69991, I69992, I69993, I69998

Cardiac Arrest and VF: I4901, 4275, 42741

Acute Renal Failure: N170, N171, N172, N178, N179, 584, 5945, 5946, 5847, 5848, 5849

Myocardial Infarction: I2101, I2102, I2111, I2113, I2114, I2119, I2121, I2129, I21A1, 410, 4100, 41000, 41001, 41002, 4101, 41010, 41011, 41012, 4102, 41020, 41021, 41022, 4103, 41030, 41031, 41032, 4104, 41040, 41041, 41042, 4105, 41050, 41051, 41052, 4106, 41060, 41061, 41062, 4107, 41070, 41071, 41072, 4108, 41080, 41081, 41082, 4109, 41090, 41091, 41092

Blood Loss Anemia: D62, 2800, 2878, 2879

Pneumonia: J189, J159, J22, 486, 481, 485, 4870, 4871, 4878, 480, 4800, 4801, 4802, 4803, 4808, 4809, 482, 4820, 4821, 4822, 4823, 48230, 48231, 48232, 48239, 4824, 48240, 48241, 48242, 48249, 4828, 48281, 48282, 48283, 48284, 48289, 4829, 483, 4830, 4831, 4838, 484, 4841, 4843, 4845, 4846, 4847, 4848, 488, 4880, 48801, 48802, 48809, 4881, 48811, 48812, 48819, 4888, 48881, 48882, 48889

Blood Transfusion: 30233N1, 990, 9900, 9901, 9902, 9903, 9904, 9905, 9906, 9907, 9908, 9909

Pulmonary Embolism: I2602, I2609, I2692, I2699, 4151, 41511, 41512, 41513, 41519

Deep Vein Thrombosis (DVT): I82401, I82402, I82403, I82409, I82411, I82412, I82413, I82419, I82421, I82422, I82423, I82429, I82431, I82432, I82433, I82439, I82441, I82442, I82443, I82491, I82492, I82493, I82499, I824Y1, I824Y2, I824Y3, I824Y9, I824Z1, I824Z2, I824Z3, I824Z4, 451, 4510, 4511, 45111, 45119, 4512, 4518, 45181, 45182, 45183, 45184, 45189, 4519

Periprosthetic Fracture: T84010A, T84011A, T84012A, T84013A, T84018A, T84019A, M9665, M96661, M96662, M96669, M96671, M96672, M96679, M9669, M9701XA, M9702XA, M9711XA, M9712XA, 99644

Periprosthetic Mechanical Complication: T84090A, T84091A, T84092A, T84093A, T84098A, T84099A, 99639, 9964, 99640, 99641, 99643, 99645, 99646, 99647, 99649

Periprosthetic Infection: T8450XA, T8451XA, T8452XA, T8453XA, T8459XA, T8454XA, 99666, 99667, 99669

Superficial SSI: T8141XA

Deep SSI: T8142XA

Wound Dehiscence: T8130XA, T8131XA, T8132XA, 99831, 99832

### Appendix A.3. Comorbidities and Corresponding ICD 9 and 10 Codes

Smoking: 3051, 30510, 30511, 30512, 30513, 30514, 30515, 30516, 30517, 30518, 30519, 64900, 64901, 64902, 64903, 64904, 98984, F17200, F17201, F17203, F17208, F17209, F17210, F17211, F17213, F17218, F17219, F17220, F17221, F17223, F17228, F17229, F17290, F17291, F17293, F17298, F17299, O99330, O99331, O99332, O99333, O99334, O99335, O99336, Z720, Z87791

Diabetes: 25000, 25001, 25002, 25003, 25010, 25011, 25012, 25013, 25020, 25021, 25022, 25023, 25030, 25031, 25032, 25033, 25040, 25041, 25042, 25043, 25050, 25051, 25052, 25053, 25060, 25061, 25062, 25063, 25070, 25071, 25072, 25073, 25080, 25081, 25082, 25083, 25090, 25091, 25092, 25093, E0800, E0801, E0802, E0803, E0804, E0805, E0806, E0807, E0808, E0809, E0810, E0811, E0812, E0813, E0814, E0815, E0816, E0817, E0818, E0819, E0820, E0821, E0822, E0823, E0824, E0825, E0826, E0827, E0828, E0829, E0830, E0831, E0832, E0833, E0834, E0835, E0836, E0837, E0838, E0839, E0840, E0841, E0842, E0843, E0844, E0845, E0846, E0847, E0848, E0849, E0850, E0851, E0852, E0853, E0854, E0855, E0856, E0857, E0858, E0859, E0860, E0861, E0862, E0863, E0864, E0865, E0866, E0867, E0868, E0869, E0870, E0871, E0872, E0873, E0874, E0875, E0876, E0877, E0878, E0879, E0880, E0881, E0882, E0883, E0884, E0885, E0886, E0887, E0888, E0889, E089, E0900, E0901, E0902, E0903, E0904, E0905, E0906, E0907, E0908, E0909, E0910, E0911, E0912, E0913, E0914, E0915, E0916, E0917, E0918, E0919, E0920, E0921, E0922, E0923, E0924, E0925, E0926, E0927, E0928, E0929, E0930, E0931, E0932, E0933, E0934, E0935, E0936, E0937, E0938, E0939, E0940, E0941, E0942, E0943, E0944, E0945, E0946, E0947, E0948, E0949, E0950, E0951, E0952, E0953, E0954, E0955, E0956, E0957, E0958, E0959, E0960, E0961, E0962, E0963, E0964, E0965, E0966, E0967, E0968, E0969, E0970, E0971, E0972, E0973, E0974, E0975, E0976, E0977, E0978, E0979, E0980, E0981, E0982, E0983, E0984, E0985, E0986, E0987, E0988, E0989, E0990, E0991, E0992, E0993, E0994, E0995, E0996, E0997, E0998, E0999, E1000, E1001, E1002, E1003, E1004, E1005, E1006, E1007, E1008, E1009, E1010, E1011, E1012, E1013, E1014, E1015, E1016, E1017, E1018, E1019, E1020, E1021, E1022, E1023, E1024, E1025, E1026, E1027, E1028, E1029, E1030, E1031, E1032, E1033, E1034, E1035, E1036, E1037, E1038, E1039, E1040, E1041, E1042, E1043, E1044, E1045, E1046, E1047, E1048, E1049, E1050, E1051, E1052, E1053, E1054, E1055, E1056, E1057, E1058, E1059, E1060, E1061, E1062, E1063, E1064, E1065, E1066, E1067, E1068, E1069, E1070, E1071, E1072, E1073, E1074, E1075, E1076, E1077, E1078, E1079, E1080, E1081, E1082, E1083, E1084, E1085, E1086, E1087, E1088, E1089, E1090, E1091, E1092, E1093, E1094, E1095, E1096, E1097, E1098, E1099, E1100, E1101, E1102, E1103, E1104, E1105, E1106, E1107, E1108, E1109, E1110, E1111, E1112, E1113, E1114, E1115, E1116, E1117, E1118, E1119, E1120, E1121, E1122, E1123, E1124, E1125, E1126, E1127, E1128, E1129, E1130, E1131, E1132, E1133, E1134, E1135, E1136, E1137, E1138, E1139, E1140, E1141, E1142, E1143, E1144, E1145, E1146, E1147, E1148, E1149, E1150, E1151, E1152, E1153, E1154, E1155, E1156, E1157, E1158, E1159, E1160, E1161, E1162, E1163, E1164, E1165, E1166, E1167, E1168, E1169, E1170, E1171, E1172, E1173, E1174, E1175, E1176, E1177, E1178, E1179, E1180, E1181, E1182, E1183, E1184, E1185, E1186, E1187, E1188, E1189, E1190, E1191, E1192, E1193, E1194, E1195, E1196, E1197, E1198, E1199

Obesity: E660, E6601, E6609, E661, E662, E668, E669, Z6830, Z6831, Z6832, Z6833, Z6834, Z6835, Z6836, Z6837, Z6838, Z6839, Z6841, Z6842, Z6843, Z6844, Z6845, 278, 2780, 27800, 27801, 27802, 27803
